# CBS and MAT2A improve methionine‐mediated DNA synthesis through SAMTOR/mTORC1/S6K1/CAD pathway during embryo implantation

**DOI:** 10.1111/cpr.12950

**Published:** 2020-11-12

**Authors:** Shuang Cai, Qianhong Ye, Xiangzhou Zeng, Guangxin Yang, Changchuan Ye, Meixia Chen, Haitao Yu, Yuming Wang, Gang Wang, Shuo Huang, Shuang Quan, Xiangfang Zeng, Shiyan Qiao

**Affiliations:** ^1^ State Key Laboratory of Animal Nutrition China Agricultural University Beijing China; ^2^ Beijing Key Laboratory of Bio‐feed Additives China Agricultural University Beijing China

## Abstract

**Objectives:**

Early pregnancy loss is a major clinical concern in animal and human reproduction, which is largely influenced by embryo implantation. The importance of methionine for embryo implantation is widely neglected.

**Materials and methods:**

We performed a series of experiments with primiparous rats fed diets containing different levels of methionine during early pregnancy to investigate the role of methionine in embryonic implantation and pregnancy outcomes, and used them to perform in vivo metabolic assessments and in vitro uterine explant culture. In addition, through transcriptome analysis and silencing the expression of cystathionine β‐synthase (CBS, the key enzyme in transsulfuration pathway) and cell adhesion assay, we measured signalling within Ishikawa, pTr and JAR cells.

**Results:**

We determined the relevance and underlying mechanism of methionine on embryo implantation. We showed that methionine deprivation sharply decreased embryo implantation sites, expression of CBS and transsulfuration pathway end products, which were reversed by maternal methionine supplementation during early pregnancy. Moreover, we found CBS improved methionine‐mediated cell proliferation and DNA synthesis by CBS inhibition or interference. In addition, transcriptome analysis also revealed that CBS influenced the signalling pathway‐associated cell proliferation and DNA synthesis, as well as a correlation between CBS and methionine adenosyltransferase 2A (MAT2A), implying that MAT2A was possibly involved in cell proliferation and DNA synthesis. Further analysis revealed that MAT2A influenced S‐adenosylmethionine receptor SAMTOR expression, and SAMTOR activated mTORC1 and its downstream S6K1 and CAD, ultimately enhancing DNA synthesis in the embryo and uterus.

**Conclusions:**

Taken together, these studies demonstrate that CBS and MAT2A improve methionine‐mediated DNA synthesis through SAMTOR/mTORC1/S6K1/CAD pathway during embryo implantation.

## Graphical abstract

Methionine deprivation sharply decreased embryo implantation sites, which were reversed by maternal methionine supplementation during early pregnancy. Methionine improved embryo proliferation and implantation through activating SAMTOR/mTORC1/S6K1/CAD signalling pathway.

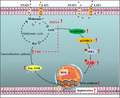

## 1 INTRODUCTION

Low fertility has become a major health issue in recent years, with the natural growth rate of the global population gradually declining. Low fertility in humans and other mammals is associated with embryonic loss during pregnancy. Embryonic loss accounts for 15%‐20% of pregnancies in humans and 30%‐40% of pregnancies in mammals, with two‐thirds of the losses occurring during early pregnancy.^1^, ^2^ In humans, the natural conception rate per menstrual cycle is approximately 30%, and 75% of all pregnancy failures are believed to be due to embryo implantation failure.^3^, ^4^ The pregnancy rate for in vitro fertilized embryos is 30%‐40%^5^ due to embryo implantation failure.^6^ In addition, more than 90% of embryos utilized in nuclear transfer technology die during the pregnancy period.^7^ Therefore, reducing early embryonic implantation failure is crucially important for improving pregnancy health in mammalian animals and humans.

The causes of embryonic implantation failure include embryo abnormalities and poor uterine receptivity,^8^, ^9^, ^10^, ^11^ which are strongly influenced by maternal nutrition status.^12^, ^13^, ^14^ Numerous studies have confirmed that the amino acid requirement increases sharply during peri‐implantation.^15^ Of particular interest, the concentration of methionine in the uterine lumen of pregnant sheep was increased by 12.39‐fold compared with that of non‐pregnant sheep on day 15 of pregnancy.^16^ In addition, methionine, histidine and lysine showed the greatest increase in the uterine lumen during the embryo elongation period (days 14‐18 of pregnancy) in dairy cattle^17^ and sheep.^17^, ^18^, ^19^ This evidence indicates that methionine plays an important role in embryo development and implantation. Methionine is an important essential amino acid with roles in many biological functions; for example, methionine is the initiating amino acid for protein synthesis in eukaryotic organisms, is a precursor for antioxidants and provides an active methyl group for methylation. Studies based on in vitro fertilized bovine embryos indicated that the disruption of methionine metabolism impaired the morula‐to‐blastocyst transition during preimplantation development (control = 40.0%, treatment = 1.1%). Additionally, the disruption of methionine metabolism caused the hypomethylation of DNA and consequently led to the altered expression of developmentally important genes.^20^ Recently, genome‐wide epigenetic and transcription profiles of in vitro cultured bovine embryos indicated that the metabolite of methionine, S‐adenosylmethionine (SAM), caused genome‐wide hypermethylation mainly in exonic regions and in CpG islands. Although differentially expressed genes were associated with the response to nutrients and developmental processes, no correspondence was found with the differentially methylated regions.^21^ Moreover, methionine supplementation caused significant changes in the embryonic transcriptome of Holstein cows, and the most significant genes were related to embryonic development and immune responses.^22^ Collectively, these studies demonstrated that methionine status influences embryo development. However, the molecular mechanisms through which maternal methionine status regulates embryo development and implantation are unexplored.

Here, we identified a critical role of CBS in improving methionine‐mediated embryo implantation and pregnancy outcomes. We discovered that methionine significantly enhanced S‐phase cell proliferation, DNA duplication and antioxidant capacity under the control of CBS. In addition, MAT2A catalysed the production of the functional metabolite SAM, which could be sensed by the receptor SAMTOR and activated mechanistic target of rapamycin complex 1 (mTORC1), ultimately regulating DNA synthesis via its downstream targets ribosomal protein S6 kinase 1 (S6K1) and carbamoyl‐phosphate synthetase 2 (CAD). Our findings revealed a novel mechanism by which methionine contributes to early embryo development and implantation by regulating the enzymes MAT2A and CBS.

## 2 MATERIALS AND METHODS

### 2.1 Animals

All procedures were approved by the China Agricultural University Animal Care and Use Committee. Mature rats (Sprague Dawley rats, 6‐8 weeks old) were purchased from Beijing Huafukang Bioscience Co., Inc, and caged in a specific pathogen‐free animal room under a controlled environment (temperature set at 23°C and a lighting schedule of 12 hours light/12 hours dark). Pregnancy was induced by co‐caging overnight, and the presence of spermatozoa in the vaginal smear was defined as day 1 of pregnancy.

The effects of dietary methionine deficiency and supplementation during early pregnancy on the embryo implantation of rats were determined. A total of 210 pregnant rats were assigned randomly into the following seven dietary groups (n = 30 per treatment group): 1. basal rodent non‐purified diet (control diet according to AIN 93; containing 0.4% methionine); 2‐5. basal rodent non‐purified diet plus 0.2%, 0.4%, 0.6% and 0.8% (wt:wt) methionine, respectively; 6. basal rodent purified diet (control diet according to AIN 93; containing 0.4% methionine); and 7. methionine‐free rodent purified diet (containing no methionine). On day 7 of pregnancy, all rats were anaesthetized by an intraperitoneal injection of 2.0% sodium pentobarbital (30 mg/kg). Blood samples were collected from the abdominal aorta, stored at room temperature for 2 hours and then centrifuged at 3500 g for 10 minutes. Serum was stored at −20°C until analysis. Uterine horns were quickly exposed, and the number of implantation sites was recorded. On day 7 of pregnancy, the implantation sites were large and could be counted without magnification.

According to the results, we found that the 1.0% methionine diet had the most significant effects on the embryo implantation of rats. Therefore, we designed the following animal experiments including only the control and 1.0% methionine groups. A total of 120 pregnant rats were randomly divided into the following four dietary groups (n = 30 per treatment group): 1. basal rodent non‐purified diet; 2. basal rodent non‐purified diet plus 0.6% (wt:wt) methionine; 3. basal rodent purified diet; and 4. methionine‐free rodent purified diet. Pregnant rats were fed the treatment diet from day 1 to day 7 of pregnancy and then fed the control diet until parturition. At birth, the litter size, live litter size, birthweight of individual rat pups and sex of rats were determined. In addition, the daily feed intake of dams and their body weights on day 1 and day 7 of pregnancy were recorded.

To further determine the possible mechanism through which methionine influences early embryo development and implantation, a total of 48 pregnant rats were randomly divided into the following two dietary groups (n = 12 per treatment group): 1. basal rodent non‐purified diet and 2. basal rodent non‐purified diet plus 0.6% (wt:wt) methionine. On day 5 and day 6 of pregnancy, 12 rats from each group were chosen randomly to be sacrificed. Blood samples were taken from the abdominal aorta and stored at room temperature, and serum was stored at −20°C until analysis. Uterine flushing was performed according to the modified methods described by Bonnamy et al^23^ Briefly, the uterine horns of each pregnant rat were exposed by laparotomy, dissected without connective tissues and clamped at the ends near the cervix. Then, 0.9% saline (0.5 mL) was flushed through one horn from the uterotubal junction, and the wash was recovered at the cervical end of the horn and then flushed through the other horn. The collected uterine washing was centrifuged at 3000 g at 4°C for 15 minutes, and the supernatant was collected and stored at −20°C until amino acid analysis. Thereafter, the uteri were frozen in liquid nitrogen quickly and then stored at −80°C.

### 2.2 Real‐time PCR analysis

Total RNA was extracted from uteri, ovaries, and JAR, Ishikawa and pTr cells using NucleoZOL reagent (Macherey‐nagel) according to the manufacturer's protocols. Total RNA quality and quantity were determined by a NanoDrop system (ND‐1000; Thermo Scientific). Reverse transcription was conducted using the PrimerScript™ RT Reagent Kit (TaKaRa). The specific primers employed for real‐time PCR were designed using Primer Premier 6.0 and Oligo 7.0 software, and the sequences of these primers are shown in Table S1. cDNA was amplified to quantify gene expression via real‐time PCR using gene‐specific primers and SYBR Green (TaKaRa). Experiments were conducted in duplicate for each sample, and the 2^−∆∆Ct^ method was used to calculate relative gene expression in different samples, with β‐actin as the reference gene.

### 2.3 Western blot analysis

Cells or frozen uteri were homogenized in RIPA lysis buffer containing protease inhibitor cocktails (Huaxingbio). After 30 minutes of incubation, the homogenates were centrifuged at 14 000 g for 15 minutes at 4°C, and the supernatant fluid was collected and stored at −80°C. Protein concentrations were determined using a BCA protein assay kit (Thermo Scientific). Equal amounts of proteins were electrophoresed (Bio‐Rad) on SDS‐polyacrylamide gels. Proteins were electrotransferred to a PVDF membrane (Millipore) and blocked with 5% non‐fat dry milk for 2 hours. Prestained protein markers were run in each gel. Samples were incubated with the primary antibodies in 5% milk‐TBST at 4°C overnight. After being washed with TBST, the membranes were incubated with a secondary antibody for 2 hours at room temperature. Chemiluminescence was detected with Western Blot Luminance Reagent (Applygen) using an ImageQuant LAS 4000 Mini System (GE Healthcare Bio‐Sciences AB, Inc) and quantified by ImageJ software (GE Healthcare Life Science). Primary antibodies against the following proteins were used: CBS, S6K1, p‐S6K1, CAD, p‐CAD, β‐actin, LAT1, SNAT1 (Santa Cruz) and SAMTOR (Atlas Antibodies).

### 2.4 Immunofluorescence analysis

Immunofluorescence analysis was performed as described previously.^24^ For immunofluorescence staining, frozen uteri samples were cryosectioned using a microtome within a cryostat (Bright Instrument Company Limited) to a thickness of 5 μmol/L onto uncoated glass microscope slides. Then, the sections were stained within 1 hour of sectioning, followed by three washes for 5 minutes in phosphate‐buffered saline (PBS, 137 mmol/L sodium chloride, 3 mmol/L potassium chloride, 8 mmol/L sodium phosphate dibasic and 3 mmol/L potassium phosphate monobasic). Monoclonal rabbit antisera to recombinant rat LAT1 (Santa Cruz) and SNAT1 (Santa Cruz) were applied to the section for 2 hours at room temperature. After primary antibody incubation, the sections were washed three times in PBS for 5 minutes each wash. Goat anti‐rabbit IgG was applied to the sections for 30 minutes at room temperature. DAPI (Sigma‐Aldrich) staining for cell nuclei was added to the secondary antibody at a 0.5 μg/mL concentration. Wide‐field image capture was completed using a Nikon E600 microscope coupled to a SPOT RT KE color three‐shot CCD camera (Diagnostic Instruments Inc).

### 2.5 Uterus explant culture

Uterine tissues were obtained within 1 hour from rats on day 5 of pregnancy. The tissues were immediately put into Dulbecco's modified Eagle's medium (DMEM) medium containing 1% penicillin‐streptomycin, and then cut into 1‐2 mm sections and cultured in vitro in 6‐well plates with 2 mL of DMEM containing 10% foetal bovine serum (FBS; Gibco) and 1% penicillin‐streptomycin. The tissues were treated with methionine at 0, 0.1, 0.2, 0.4, 0.8‐ or 1.6‐mmol/L methionine for 24 hours in a 37°C incubator. Then, the tissues were collected to measure the glutathione (GSH) concentration.

### 2.6 Chemical analysis

The free amino acid concentrations in serum and uterine flushing fluid were measured by reversed‐phase high‐performance liquid chromatography (HPLC) as previously described,^25^ with minor changes. Briefly, the amino acids were derivatized to fluorescent products by an automated reaction of a 25 μL sample with an equal volume of o‐phthaldialdehyde (OPA) containing 2 μL/mL 2‐mercaptoethanol. The flow rate through the column was 1.4 mL/min, and the column temperature was maintained at 25°C. The two solvents required to generate the elution gradient were a 1:4 and 4:1 (v/v) ratio of methanol:sodium acetate.

Estradiol and progesterone in serum were determined by radioimmunoassays with an E2‐17β (HY‐10029) RIA Kit and a P (HY‐031) RIA Kit (Sino‐UK Bio) according to the manufacturer's protocols.

### 2.7 ELISAs

The serum concentrations of enzymes involved in the methionine metabolism pathway, adenosylmethionine synthetase (SAMs), DNA methyltransferases (Dnmts), CBS, cystathionine gamma lyase (CSE) and methionine synthase (MS) were determined using commercially available ELISA Kits (Cusabio Biotech Co., Ltd.; Meibao Biotech Co.). All samples were analysed in duplicate in a single assay.

The GSH concentrations in serum and uterine tissues were measured using a commercially available ELISA Kit (Nanjing Jiancheng Biotech Co.). All samples were analysed in duplicate in a single assay.

### 2.8 Non‐targeted metabolomic analysis

We used methanol to extract both intracellular and media/extracellular metabolites. Serum samples (200 μL) were centrifuged at 14 000 g for 20 minutes at 4°C. The supernatant was transferred and mixed with four volumes of 100% methanol. The supernatant media were incubated at −80°C for 2 hours. The samples were then centrifuged at 14 000 g for 10 minutes at 4°C. The metabolites containing supernatants were transferred to new tubes on dry ice and then dried to pellets using a SpeedVac/lyophilizer with no heat. Extracts were analysed by HPLC‐MS, as described previously.^26^

### 2.9 Cell lines and cell culture

Ishikawa cells (human endometrial epithelial cell line), JAR cells (human trophoblastic cell line) and pTr cells (porcine trophoblast cells) were obtained from the European Collection of Authenticated Cell Cultures (ECACC) and the American Type Culture Collection (ATCC). Ishikawa and JAR cells were cultured in RPMI 1640 medium (Gibco) supplemented with 10% FBS, while pTr cells were maintained in DMEM/F12 (Gibco) supplemented with 10% FBS and 1% ITS. The cells were maintained at 37°C in a humidified atmosphere with 5% CO_2_.

### 2.10 Cell proliferation analysis

After reaching 80% to 90% confluence, the cells were starved in amino acid–free medium with Earle's balanced salts (EBSS; Sigma‐Aldrich) supplemented with a vitamin mixture (Sigma‐Aldrich) for 2 hours or in customized methionine‐free medium (Sigma‐Aldrich) overnight. After starvation, the cells were used for the following experiments.

The cells were cultured in methionine‐free medium supplemented with 0, 0.031, 0.062, 0.125, 0.25, 0.50, 1.0, 2.0 or 4.0 mmol/L methionine (Sigma‐Aldrich). After 12 hours, the proliferation of the cells was analysed. The time‐dependent change (0, 6, 12, 18, 24, 30 and 36 hours) in cell proliferation was determined. Based on the finding that JAR cell proliferation increased significantly after treatment with 0.25 mmol/L methionine for 12 hours, we added 0.125, 0.25 and 0.5 mmol/L ethionine and O‐(carboxymethyl) hydroxylamine hemi‐hydrochloride (AOAA) in 0.25 mmol/L methionine medium and cultured the cells for 12 hours to determine the change in cell proliferation. In addition, we treated JAR cells with 0‐ or 0.25‐mmol/L methionine, a mixture of 0.25 mmol/L methionine and 0.25 mmol/L ethionine, or a mixture of 0.25 mmol/L methionine and 0.25 mmol/L ethionine for 12 hours and then analysed the cell cycle and intracellular ROS. Additionally, rapamycin was used to inhibit the activity of mTORC1. We pre‐treated cells with rapamycin for 12 hours. Afterwards, the culture medium was discarded and cells were rescued with methionine and SAM. Cells were collected for Western blot. Ishikawa and pTr cells were treated similarly to verify these results. All experiments were repeated at least three times.

In addition, JAR cells were cultured in EBSS medium supplemented with 0, 0.125, 0.25, 0.50, 1.0, 2.0 or 4.0 mmol/L methionine. After 2 hours, the proliferation of the cells was measured. The time‐dependent change (0, 0.5, 1.0, 1.5, 2.0, 2.5 and 3.0 hours) in cell proliferation was determined. According to the result that JAR cell proliferation increased significantly after treatment with 2.0 mmol/L methionine for 2.5 hours, we supplemented 1.5, 2.0 and 2.5 mmol/L ethionine and AOAA in 2.5 mmol/L methionine medium, cultured the cells for 2.5 hours and determined the change in cell proliferation. Moreover, we treated JAR cells with 0‐ or 2.0‐mmol/L methionine, a mixture of 2.0 mmol/L methionine and 2.0 mmol/L ethionine, or a mixture of 2.0 mmol/L methionine and 2.0 mmol/L ethionine for 2.5 hours and then analysed the cell cycle and intracellular ROS. Ishikawa and pTr cells were treated similarly to verify these results. All experiments were repeated at least three times.

Cell proliferation was measured using a CCK‐8 Kit (Dojindo) according to the manufacturer's protocols. The incremental proliferation index (PI) of these cells was calculated according to PI = [(Ad − Ab)/(Aw − Ab)] × 100%, where Ad represents the optical density (OD) values after the treatment, Aw represents the OD values without the treatment, and Ab represents the OD values of the blank wells (only the culture medium, no cells).

### 2.11 Intracellular ROS analysis

Intracellular ROS were measured using DCFH‐FA (Nanjing Jiancheng) according to the manufacturer's protocols.

### 2.12 Flow cytometry analysis

Cells were harvested and fixed in 70% ethanol (added dropwise while vortexing) and stored at 4°C overnight. The cells were centrifuged at 200 *g* for 5 minutes at 4°C and then washed once with PBS, centrifuged again and stained with staining solution (2 mg/mL propidium iodide) (Thermo Scientific) and 10 mg/mL RNase A (Sigma‐Aldrich) for 30 minutes at 37°C. Fluorescence‐activated cell sorting analysis was carried out on a FACSCalibur Flow Cytometer (BD Biosciences). For each condition, at least 5000 events were recorded, and data analysis was performed using FlowJo software.

### 2.13 Cell adhesion assay

Embryo adhesion was performed as previously described^27^ with minor changes. Briefly, Ishikawa and JAR cells were grown on 6‐well plates. JAR cells were stained with carboxyfluorescein succinimidyl ester (CFSE; Invitrogen) for 1 hour before the adhesion assay. The stained cells were plated into Ishikawa cell monolayers in JAR cell culture medium. After 1 hour, unattached JAR cells were removed, and the attached cells were gently washed with PBS three times. The cells were then photographed under a fluorescence microscope or analysed using flow cytometry. An equal amount of stained JAR cells was plated in three blank wells. The adhesion rate was calculated as the percentage of attached JAR cells. All experiments were replicated three times.

### 2.14 RNA interference

Small interfering RNA (siRNA) was used for CBS and MAT2A knockdown, and the siRNA sequences are listed in Table S2. After reaching 70% to 90% confluence, cells were used for siRNA transfection. siRNA transfection was performed according to the manufacturer's protocols. Ishikawa, JAR and pTr cells were cotransfected with either targeting siRNA or control siRNA using Lipofectamine 3000 Reagent for 48 hours. Subsequent real‐time PCR analyses were performed to validate the knockdown efficacy of siRNA.

### 2.15 Transcriptome analysis

Total RNA was isolated from pTr cells. The quality and concentration of RNA were determined using a NanoPhotometer (Implen), a Qubit R3.0 Fluorometer (Life Technologies) and an Agilent 2100 RNA Nano 6000 Assay Kit (Agilent Technologies). Libraries were prepared according to the NEBNext R Ultra™ RNA Library Prep Kit for Illumina (#E7530L, NEB). To guarantee the data quality, the useful Perl script was used to filter the original data. The reference genomes and the annotation file were downloaded from the Ensembl database. Bowtie2 v2.2.3 was used to build the genome index, and clean data were then aligned to the reference genome using HISAT2 v2.1.0. The reads count for each gene in each sample was counted by HTSeq v0.6.0, and fragments per kilobase million mapped reads (FPKM) was then calculated to estimate the expression level of the genes in each sample. DEGseq was used for differential gene expression analysis between two samples with non‐biological replicates. A *P*‐value was assigned to each gene and adjusted by Benjamini and Hochberg's approach to control for the false discovery rate.

### 2.16 Statistical analysis

Two‐tailed Student's tests were used for single comparisons and analysis of variance (ANOVA) with LSD tests for multiple comparisons. *P*‐values below .05 were considered significant. Statistical analysis was performed using SPSS, and graphic was constructed with GraphPad Prism 6 software. Data are presented as the mean ± SEM unless stated otherwise. Non‐targeted metabolic fingerprinting statistical analysis was performed on the identified polar metabolites. PLSDA and pathway analysis were completed using MetaboAnalyst (http://www.methionineaboanalyst.ca). Enrichment cluster, pathway cluster and protein network analyses were performed using Metascape (http://metascape.org/gp/index.html#/main/step1) according to Zhou et al^28^

## 3 RESULTS

### 3.1 Maternal methionine levels impacted embryonic implantation and pregnancy outcomes

**FIGURE 1:**
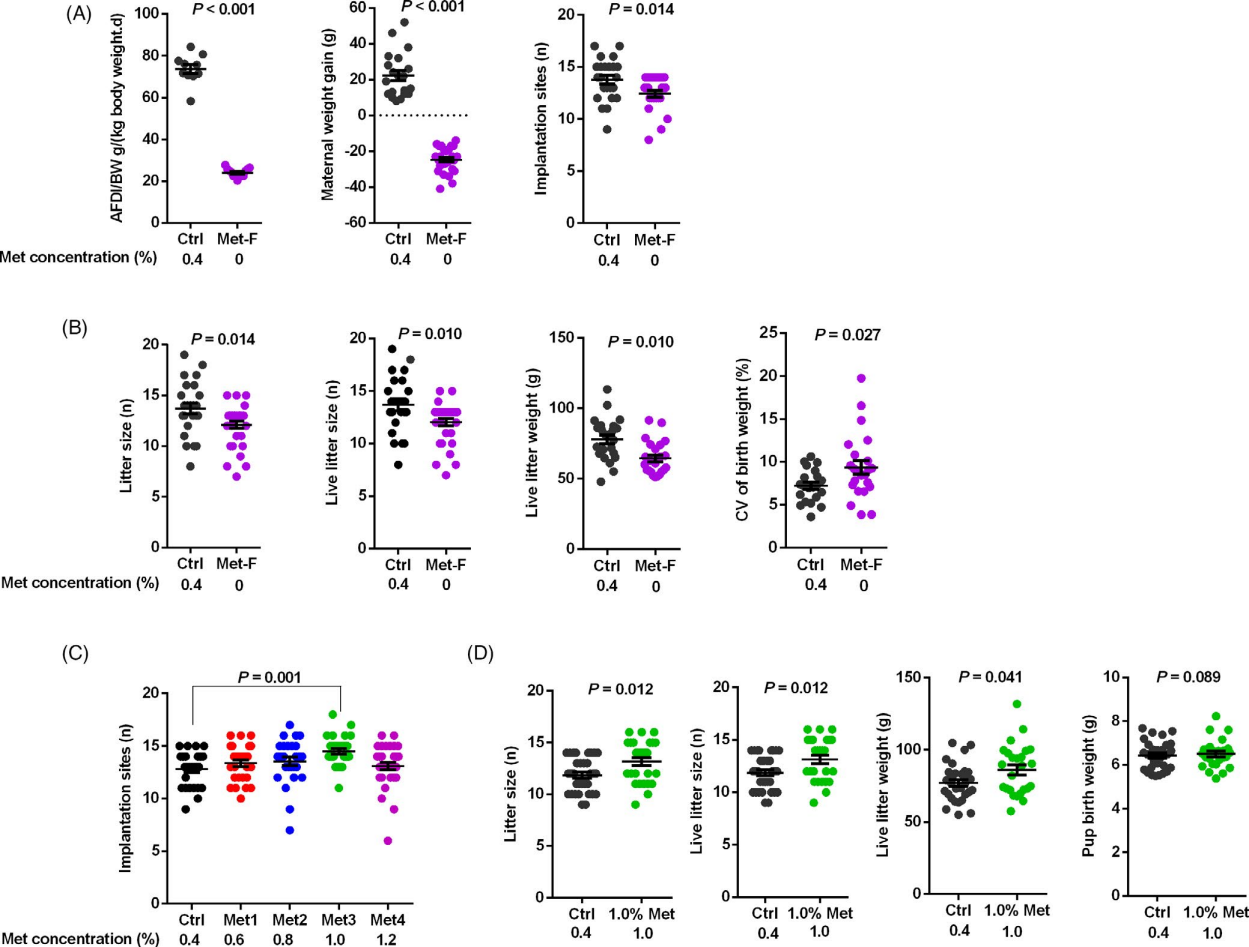
Methionine levels impacted embryonic implantation and pregnancy outcomes. A, The methionine‐free diet significantly decreased AFDI/BW, maternal weight gain and the number of implantation sites in rats. B, The methionine‐free diet significantly decreased litter size, live litter size and live litter weight and increased the coefficient of variance (CV) of the birthweight of rat pups born alive. C, The diet containing 1.0% methionine significantly increased the number of implantation sites. D, Litter size, live litter size and live litter weight were increased in 1.0% methionine group. n = 30

To determine the role of methionine in embryonic implantation and pregnancy outcomes, we performed a series of experiments with rats fed a methionine‐free diet (Met‐F, containing no methionine) or diets containing different levels of methionine during early pregnancy. The results showed that compared with the control diet (Ctrl, containing 0.4% methionine), the methionine‐free diet significantly decreased average daily feed intake/body weight (AFDI/BW), maternal weight gain and the number of implantation sites in rats (Figure 1A). Additionally, the methionine‐free diet significantly decreased litter size, live litter size and live litter weight, while the coefficient of variance (CV) of the birthweight of rat pups born alive was increased in methionine‐free diet group compared with that of the control group (Figure 1B).

Notably, a diet containing 1.0% methionine increased the number of implantation sites (Figure 1C). Additionally, compared with the control diet, the diet containing 1.0% methionine significantly increased the litter size, live litter size and live litter weight. Moreover, the average birthweight of the live pups tended to increase when a diet containing 1.0% methionine was provided during early pregnancy (Figure 1D). Collectively, these observations suggested that proper dietary methionine levels had a positive influence on embryonic implantation during early pregnancy, which benefited later pregnancy outcomes.

### 3.2 Maternal methionine levels modulated the metabolism of serum amino acids

**FIGURE 2:**
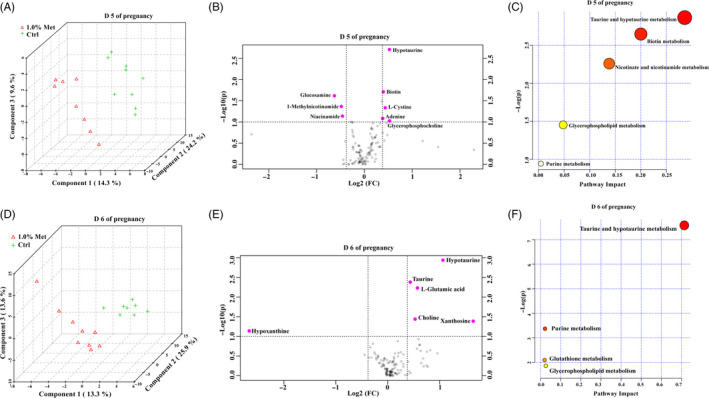
Maternal methionine levels modulated the metabolism of serum amino acids. A, PLSDA of serum on day 5 of pregnancy. B, Important features selected by a volcano plot for serum on day 5 of pregnancy. C, Differential pathways in serum on day 5 of pregnancy. D, PLSDA of serum on day 6 of pregnancy. E, Important features selected by a volcano plot for serum on day 6 of pregnancy. F, Differential pathways in serum on day 6 of pregnancy. n = 9

To further determine how methionine contributes to embryonic implantation, we measured serum metabolites on day 5 and day 6 of pregnancy. A significant difference was observed between the 1.0% methionine and control groups on day 5 of pregnancy (Figure 2A). According to the criteria of FC > 1.30 and *P* < .1, eight metabolites were selected from a volcano plot (Figure 2B). Interestingly, five metabolites were upregulated and three were downregulated in the 1.0% methionine group compared with the control group. Moreover, five pathways associated with the differentially expressed metabolites were identified by biochemical pathway analysis, namely biotin metabolism, taurine and hypotaurine metabolism, nicotinate and nicotinamide metabolism, glycerophospholipid metabolism and purine metabolism (Figure 2C).

Similarly, a significant difference was observed between the 1.0% methionine and control groups on day 6 of pregnancy (Figure 2D). Six metabolites were upregulated, while three were downregulated in the 1.0% methionine group compared with the control group (Figure 2E). Additionally, we identified four pathways that differed between the 1.0% methionine and control groups (Figure 2F): taurine and hypotaurine metabolism, glycerophospholipid metabolism, purine metabolism and GSH metabolism. Taken together, these findings provide evidence that maternal methionine levels can influence the metabolism of biotin, taurine, hypotaurine, nicotinate, nicotinamide, glycerophospholipids, purines and GSH.

### 3.3 Methionine levels influenced circulating progesterone (P_4_) and oestradiol (E_2_)

**FIGURE 3:**
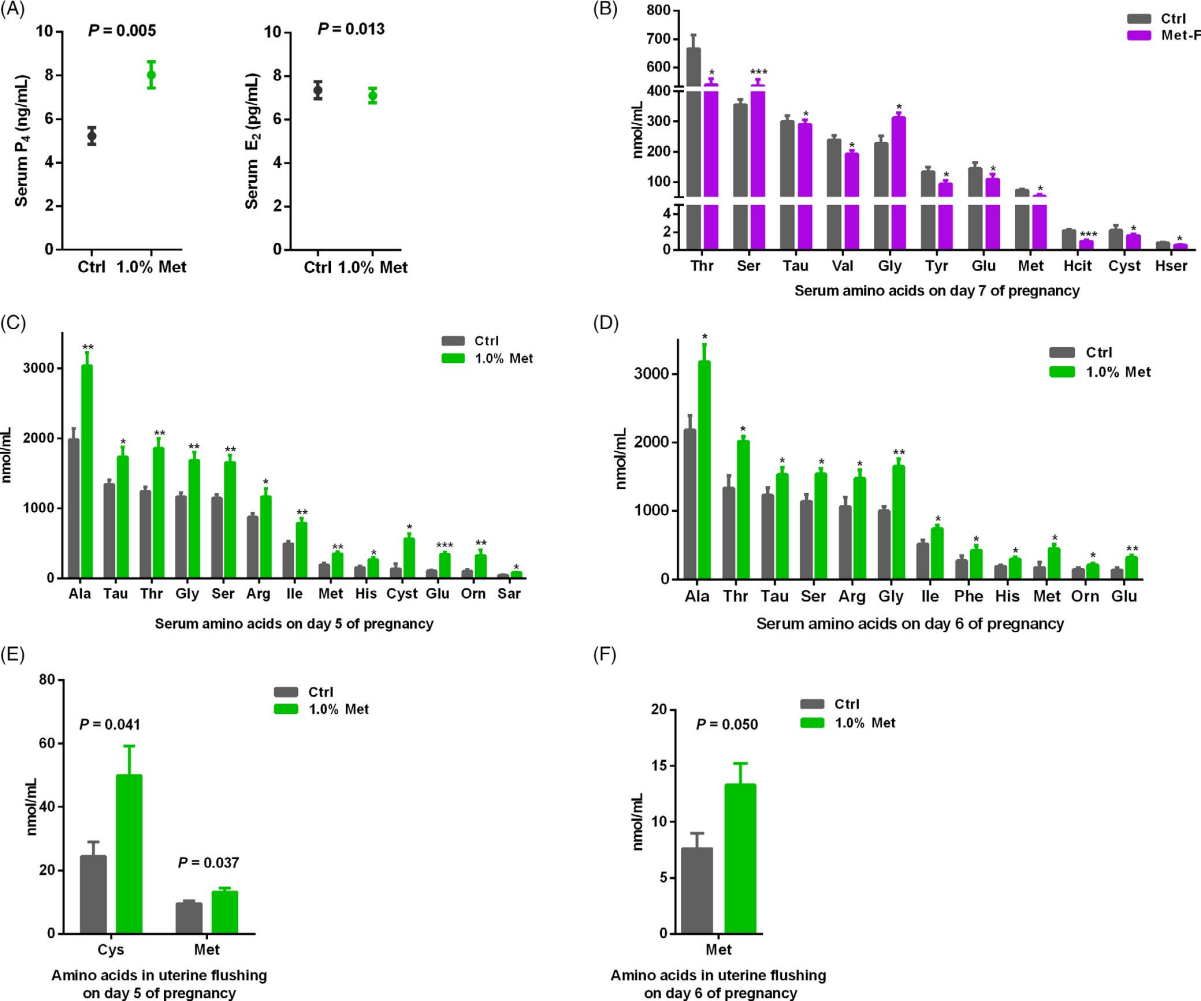
Methionine levels influenced circulating P_4_ and E_2_ and the profiles of free amino acids in serum and uterine flushing fluid. A, The levels of serum P_4_ and E_2_ on day 5 of pregnancy. B, Concentrations of amino acids in serum on day 7, (C) day 5 and (D) day 6 of pregnancy. E, Concentrations of amino acids in uterine flushing fluid on day 5 and F, day 6 of pregnancy. Each bar represented the concentration in rats with n = 12 replicates ± SE. Standard three‐letter abbreviations were used for amino acids. ***P < .001; **P < .01; *P < .05

The ovary produces the hormones P_4_ and E_2_, which prime the uterus for embryo attachment and implantation and are necessary for decidualization.^29^ Therefore, we measured P_4_ and E_2_ in pregnant rats during the peri‐implantation period and found that diets containing 1.0% methionine resulted in a significantly higher level of serum P_4_ and a lower level of serum E_2_ than the control diet on day 5 of pregnancy (Figure 3A).

### 3.4 Methionine levels modulated the profile of free amino acids in the serum and uterine flushing fluid

To test whether maternal methionine levels affected the serum and intrauterine environment during peri‐implantation, we examined the concentration of free amino acids in the serum and uterine flushing fluid. The methionine‐free diet markedly decreased the serum concentrations of homoserine, taurine, glutamate, methionine, homocitrulline, cystathionine, valine, tyrosine and threonine but increased the serum concentrations of glycine and serine on day 7 of pregnancy (Figure 3B). In contrast, a diet containing 1.0% methionine drastically enhanced the serum concentrations of taurine, threonine, serine, glutamate, glycine, alanine, sarcosine, methionine, isoleucine, histidine, cystathionine, ornithine and arginine on day 5 of pregnancy (Figure 3C). Similarly, compared with the control diet, the diet containing 1.0% methionine significantly increased the serum concentrations of taurine, threonine, serine, glutamate, glycine, alanine, phenylalanine, methionine, isoleucine, histidine, ornithine and arginine on day 6 of pregnancy (Figure 3D). These results suggested that dietary methionine levels changed the profile of free amino acids in the serum, including essential and non‐essential amino acids. In uterine flushing fluid, compared with the control diet, the diet containing 1.0% methionine significantly increased the concentrations of cysteine and methionine on day 5 of pregnancy (Figure 3E). On day 6 of pregnancy, only the concentration of methionine was increased in uterine flushing fluid in the 1.0% methionine group compared with the control group (Figure 3F).

### 3.5 Methionine levels influenced the methionine transsulphuration pathway

**FIGURE 4:**
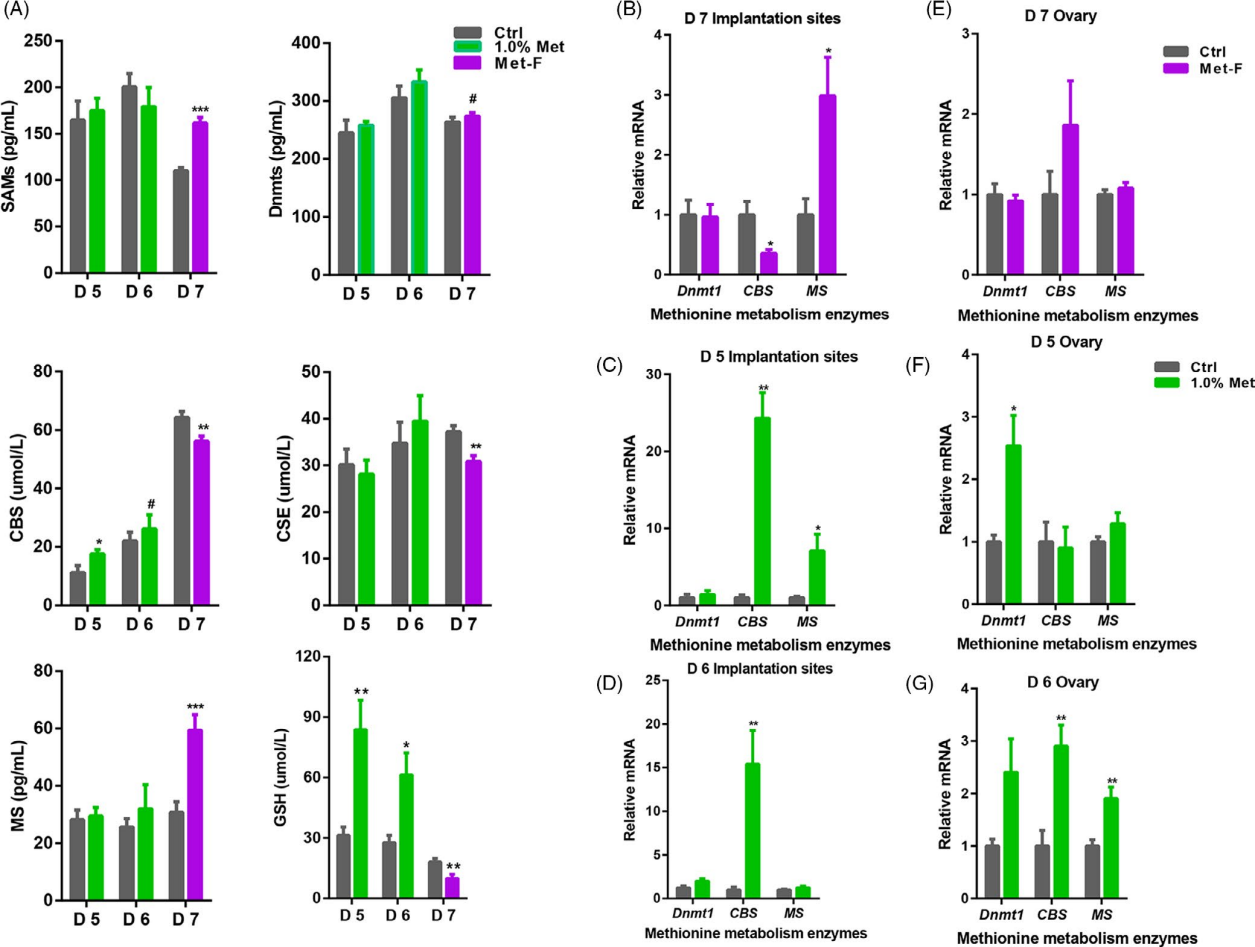
Methionine levels influenced methionine metabolism. A, Concentrations of key enzymes and end products involved in methionine metabolism in serum during peri‐implantation. B, Relative mRNA expression of key enzymes involved in methionine metabolism in implantation sites on day 7, (C) day 5 and (D) day 6 of pregnancy. E, Relative mRNA expression of key enzymes involved in methionine metabolism in ovarian tissues on day 7, (F) day 5 and (G) day 6 of pregnancy. Each bar represented the results in rats with n = 12 replicates ± SE. ***P < .001; **P < .01; *P < .05

The methionine metabolism pathway consists of two parts: (a) the ubiquitous methionine cycle, referred to as the transmethylation cycle or the ‘activated’ methionine cycle, and (b) the transsulfuration pathway.^30^ Five important enzymes, namely SAMs, Dnmts, CBS, CSE and MS, are involved in methionine metabolism. To identify whether maternal methionine content influenced methionine metabolism, we measured the concentrations of these five enzymes in serum. Compared with the control diet, the methionine‐free diet significantly increased the serum concentrations of SAMs and MS but decreased the serum concentrations of CBS and CSE on day 7 of pregnancy (Figure 4A). Notably, compared with the control diet, the diet with 1.0% methionine significantly increased the serum concentration of CBS on day 5 of pregnancy. However, the 1.0% methionine diet did not affect the serum enzyme concentration on day 6 of pregnancy, and only CBS tended to be higher in the 1.0% methionine group than in the control diet group (Figure 4A).

The uterus and ovary are important reproductive tissues. To determine the possible target tissue, we measured the relative mRNA levels of key enzymes (*Dnmt1, Dnmt3b, CBS, CSE* and *MS*) in the implantation sites and ovaries. The expression of *Dnmt3b* and *CSE* was not detected (Ct value > 35) in the implantation sites and ovaries. However, the expression of *MS* was significantly increased, and the expression of *CBS* was significantly decreased on day 7 of pregnancy in the methionine‐free group (Figure 4B). The expression levels of *CBS* and *MS* were significantly increased on day 5 of pregnancy in the 1.0% methionine group compared with the control group (Figure 4C), while only *CBS* expression was significantly increased on day 6 of pregnancy compared with that in the control group (Figure 4D). In ovaries, the expression of *Dnmt1*, *CBS* and *MS* in the methionine‐free and control groups was not different (Figure 4E). However, the expression of *Dnmt1* was significantly higher in the 1.0% methionine group than in the control group on day 5 of pregnancy (Figure 4F), while the expression of *CBS* and *MS* was significantly higher on day 6 of pregnancy (Figure 4G).

CBS is involved in the transsulfuration pathway. To evaluate whether the methionine transsulfuration pathway was changed, we measured the concentrations of end products of the methionine transsulfuration pathway, including taurine and GSH. Compared with the control group, the methionine‐free diet significantly decreased serum taurine and GSH concentrations on day 7 of pregnancy. However, compared with the control group, the 1.0% methionine diet significantly increased the concentrations of taurine and GSH in the serum of rats on day 5 and day 6 of pregnancy (Figure 4A). Additionally, the concentration of GSH in the implantation sites of rats fed a 1.0% methionine diet was increased compared with that of rats fed a control diet on day 5 and day 6 of pregnancy (Figure S1). Taken together, our findings revealed that the increase in dietary methionine levels enhanced the maternal transsulfuration pathway during early pregnancy in rats.

### 3.6 Methionine levels influenced methionine transporter expression in the implantation sites and ovaries

Amino acid transporters are critical for the supply of crucial nutrients with inherent signalling properties that sometimes even initiate signalling themselves.^31^ To determine whether methionine supplementation influenced the expression of methionine transporters, we measured the expression levels of seven main methionine transporters in implantation sites and ovaries, including S*NAT1*, *SNAT2*, *LAT1*, *LAT2*, *B^0^AT1*, *Asc1* and *TAUT,* on day 5 and day 6 of pregnancy. In the 1.0% methionine group, *SNAT1* and *LAT1* transporter levels were significantly higher in implantation sites on day 5 of pregnancy, with a fold change of approximately 1.5 compared with the control group (Figure S2A), while the expression of the *SNAT1*, *SNAT2* and *LAT1* transporters was significantly increased in implantation sites on day 6 of pregnancy, with a fold change more than 10 for *SNAT1* and *LAT1* (Figure S2B). Consistently, the levels of *SNAT1*, *SNAT2*, *LAT1*, *LAT2* and *Asc1* transporters in the 1.0% methionine group were significantly greater than those in the control group on day 5 of pregnancy in ovarian tissue (Figure S2C), while only the expression of *LAT1* was significantly greater on day 6 of pregnancy (Figure S2D). Further confirming these results, 1.0% dietary methionine significantly increased the relative protein abundance of SNAT1 and LAT1 in implantation sites on day 6 of pregnancy (Figure S2E). Moreover, immunofluorescence analysis revealed that LAT1 and SNAT1 were localized to both the nucleus and cytoplasm, although primarily in the nucleus, in the rat uterus on day 6 of pregnancy (Figure S2F).

The implantation sites included uterine tissues and embryos. To further explore the exact effects of methionine on the uterus and embryo, we separated rat uterine tissues on day 5 of pregnancy for in vitro uterine explant culture and treated the cultures with different levels of methionine. The results showed that compared with no methionine, 0.4 mmol/L methionine significantly increased the GSH concentration (Figure S3A). Additionally, the expression of key enzymes involved in methionine metabolism (*Dnmt1*, *CBS*, *CSE and MS*) was significantly increased in the 0.4 mmol/L methionine treatment group compared with the methionine‐free treatment group (Figure S3B).

### 3.7 Methionine levels modulated the transcriptome of pTr cells

**FIGURE 5:**
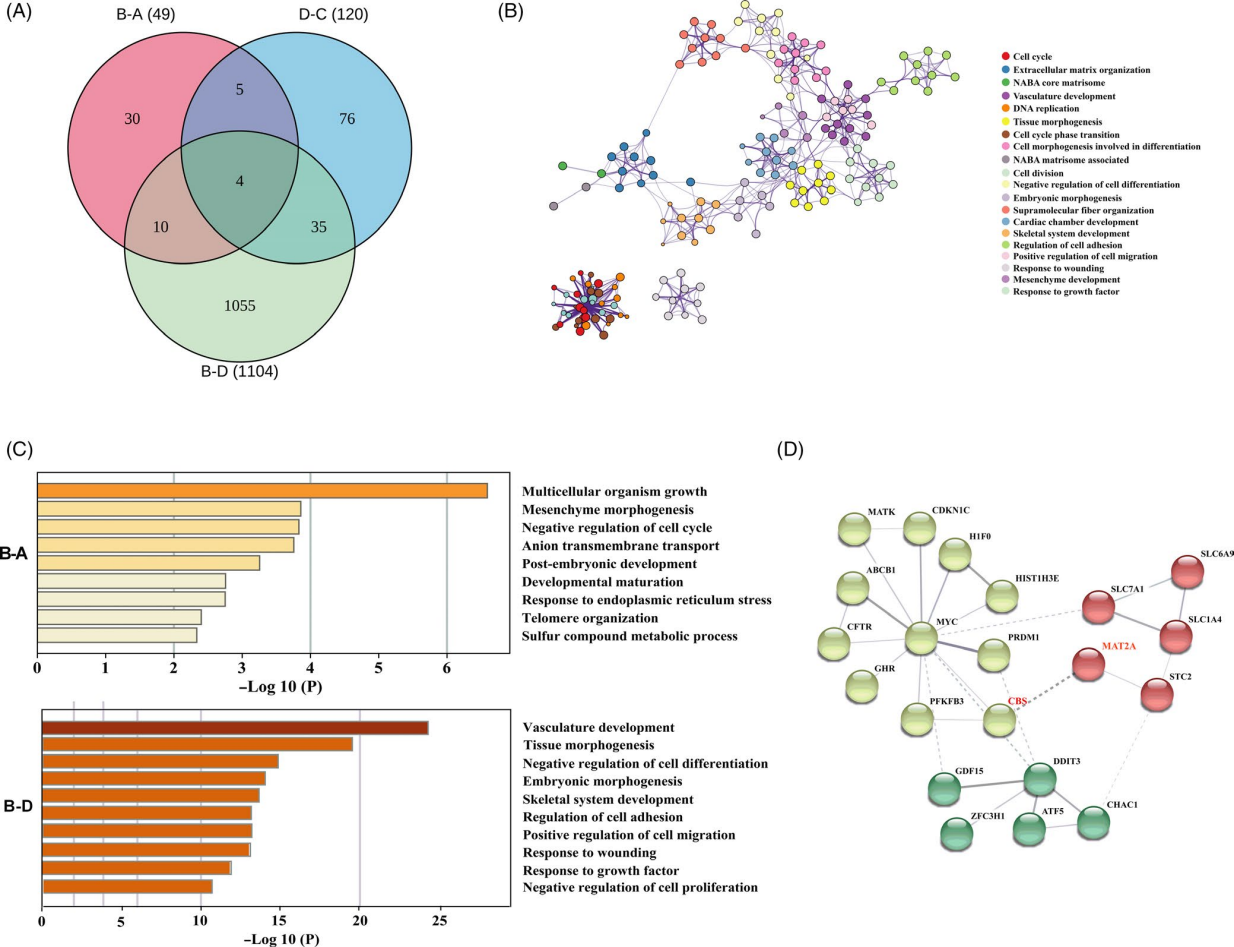
Methionine levels modulated the transcriptome of pTr cells. A, Venn diagram comparing unique or common transcripts among the methionine treatment group, CBS silencing group and methionine‐free group. B, A network plot showing the subset of enriched terms. Terms with the best *P*‐values from each of the 20 clusters were selected, with the constraint no more than 10 terms per cluster. C, Metascape bar graph showing the top non‐redundant enriched clusters. D, Protein interaction network based on selected differentially expressed genes. n = 3. A: methionine‐free group; B: methionine treatment group; C: CBS^−/−^, − methionine; D: CBS^−/−^, + methionine

To further explore the effects of methionine on embryo development, transcriptome profiling of pTr cells treated with methionine and CBS silencing was conducted. The results revealed that 49 (20 increased and 29 decreased) genes were differentially expressed in methionine‐treated cells compared with methionine‐free cells. In CBS‐silenced cells, 120 (88 increased and 32 decreased) genes were differentially expressed in methionine‐treated cells compared with methionine‐free cells. In methionine‐treated cells, 1104 (642 increased 462 and decreased) genes were differentially expressed in CBS‐expressing cells compared with CBS‐silenced cells (Figure 5A). Functional analysis of all of the differentially expressed genes was performed using Gene Ontology (GO) terms, KEGG pathways, Reactome gene sets, canonical pathways and CORUM complexes. The results revealed that methionine supplementation mainly influenced the cell cycle, DNA replication and cell cycle phase transition, while CBS mainly influenced cell adhesion, embryonic morphogenesis, extracellular matrix organization and cell differentiation (Figure 5B; Figure S4A).

To understand the specific roles of methionine and the critical enzyme CBS in more detail, enrichment clustering and protein network analysis of the methionine‐ and CBS‐treated groups were conducted. Compared with the methionine‐free condition, methionine supplementation significantly impacted multicellular organism growth, cell cycle, anion transmembrane transport, post‐embryonic development and endoplasmic reticulum stress (Figure 5C). In the methionine supplementation groups, CBS silencing treatment significantly impacted cell differentiation and proliferation, embryonic morphogenesis and cell adhesion (Figure 5C), suggesting that methionine improved embryo development and implantation through enhanced CBS expression. In addition, in CBS‐silenced cells, methionine supplementation significantly impacted DNA replication, DNA repair and the cell cycle (Figure S4B), indicating an influence of methionine on DNA replication in pTr cells through a pathway other than the regulation of CBS.

Functional analysis revealed that methionine supplementation improved embryonic growth and endoplasmic reticulum stress through the regulation of CBS and other pathways. Notably, the expression of MAT2A was substantially reduced in CBS‐silenced cells. We then analysed the protein interaction network based on selected differentially expressed genes and found an interaction between MAT2A and CBS (Figure 5D).

### 3.8 Methionine enhanced the antioxidant capacity in JAR, Ishikawa and pTr cells

**FIGURE 6:**
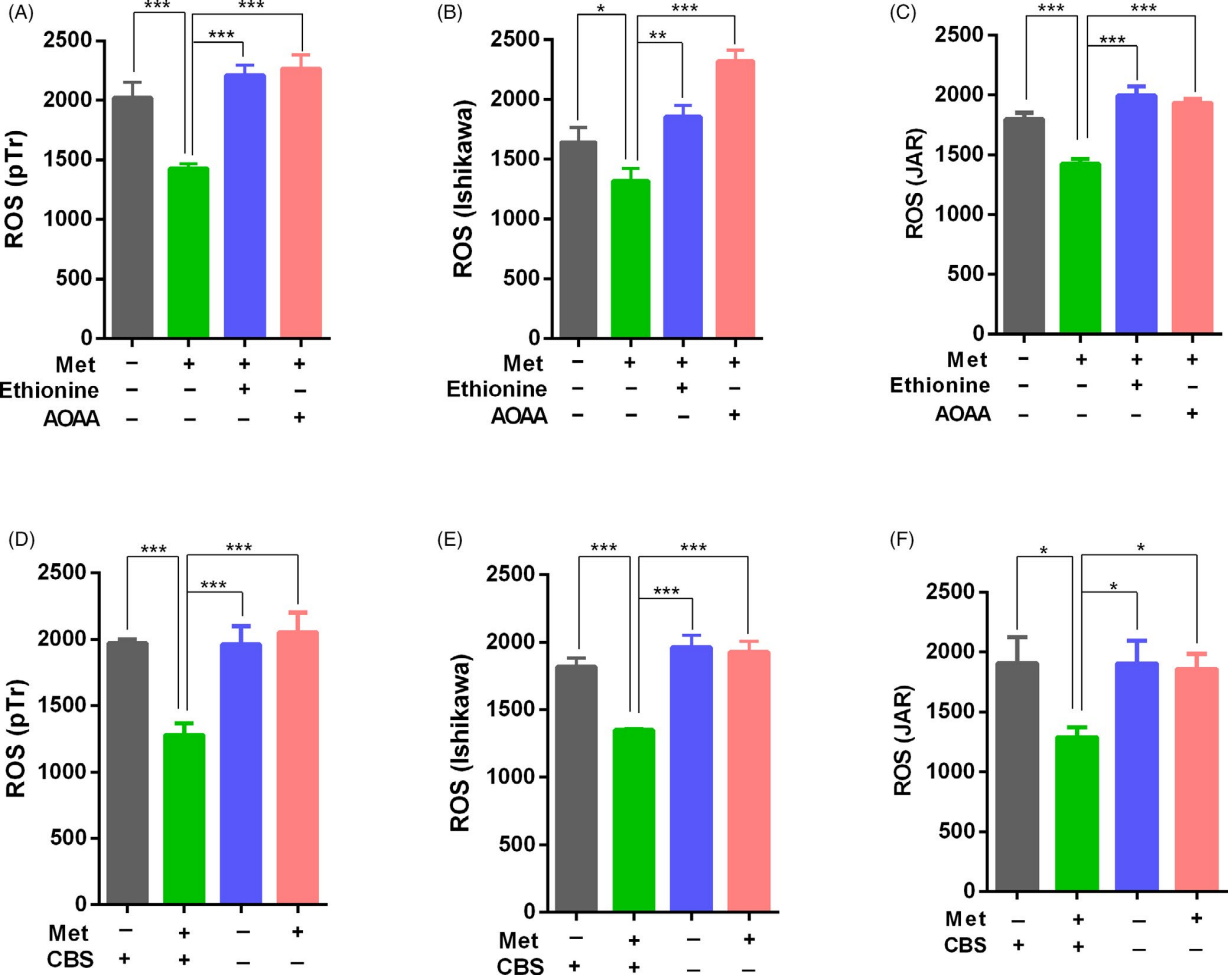
Methionine levels impacted antioxidant capacity in JAR, Ishikawa and pTr cells. A, pTr cells, (B) Ishikawa cells and (C) JAR cells were starved overnight in serum and methionine‐free medium and then treated with methionine, ethionine and AOAA. Cells were collected to detect intracellular ROS. D, Intracellular ROS in CBS‐silenced and CBS‐expressing pTr cells, (E) Ishikawa cells and (F) JAR cells treated with methionine. n = 3. ***P < .001; **P < .01; *P < .05

Oxidative stress is involved in the aetiology of defective embryo development. ROS can alter most types of cellular molecules and induce developmental block or retardation.^32^ GSH appears to be the main non‐enzymatic defence system against ROS in embryos.^33^ Metabolic pathway analysis showed that methionine supplementation significantly enhanced the metabolism of GSH in rat serum during peri‐implantation. In addition, our transcriptome functional analysis revealed that methionine supplementation influenced endoplasmic reticulum stress. To confirm the effects of methionine on oxidative stress, we measured ROS concentrations in JAR, Ishikawa and pTr cells treated with methionine, ethionine, AOAA and CBS silencing. Ethionine is a structural analog of methionine, with an ethyl group in place of the methyl group. Ethionine competitively inhibits many biochemical reactions involving methionine.^34^ AOAA, a CBS inhibitor, can inhibit the transsulphuration pathway of methionine.^35^ Methionine supplementation significantly decreased intracellular ROS, and this decrease was attenuated by ethionine and AOAA supplementation and by CBS silencing. Ishikawa, JAR and pTr cells showed similar results (Figure 6). These data suggested that methionine enhanced the cell antioxidant capacity of cells through the regulation of CBS.

### 3.9 Methionine promoted the proliferation of JAR, Ishikawa and pTr cells

**FIGURE 7:**
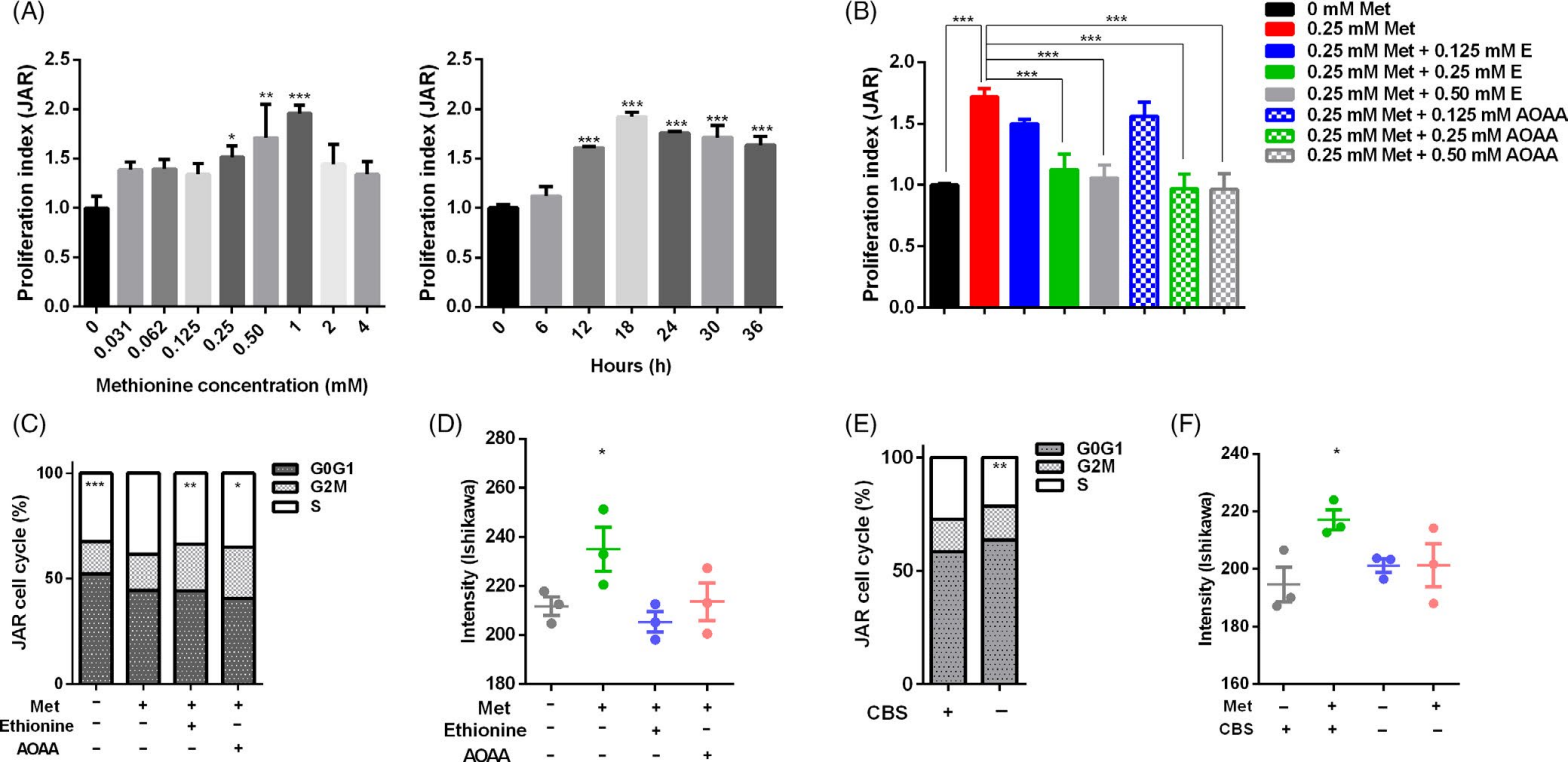
Methionine levels impacted cell proliferation in JAR, Ishikawa and pTr cells. A, JAR cells were starved overnight in serum and methionine‐free medium and then treated with different doses of methionine for 12 h. The proliferation index was detected by the CCK‐8 method. After that, JAR cells during different durations were treated with the optimal methionine concentration, according to the cell proliferation index. B, JAR cells were treated with methionine, ethionine and AOAA. The proliferation index was detected by CCK‐8 analysis. C, JAR cells were treated with methionine, ethionine and AOAA. Cells were collected for flow cytometry assays. D, Ishikawa cells were treated with methionine, ethionine and AOAA. Cells were collected to detect DNA synthesis. E, CBS was silenced in JAR cells and treated with methionine. Cells were collected for flow cytometry assays. F, CBS was silenced in Ishikawa cells and treated with methionine. Cells were collected to detect DNA synthesis. n = 3. ***P < .001; **P < .01; *P < .05

Based on the metabolome data, purine metabolism was significantly impacted throughout the peri‐implantation period. Purine is necessary for the development and survival of various cells.^36^ Purine nucleotides play critical roles in DNA and RNA synthesis as well as in membrane lipid biosynthesis and protein glycosylation.^37^ Our transcriptome data showed that supplementation significantly impacted embryonic proliferation. To identify the mechanism by which methionine regulated cell proliferation, we assessed the proliferation of JAR, Ishikawa and pTr cells treated with different doses of methionine in the culture medium. The results showed that the proliferation of JAR, Ishikawa and pTr cells was significantly promoted following treatment with appropriate methionine levels in customized methionine‐free 1640 medium (Figure 7A; Figure S5A,B), and in medium with EBSS and a 1% vitamin solution (Figure S5C‐E).

To confirm whether methionine could promote cell proliferation through the modulation of the transsulphuration pathway, we treated JAR, Ishikawa and pTr cells with ethionine or AOAA. As shown in Figure 7B, in the customized methionine‐free 1640 medium, 0.25 and 0.5 mmol/L ethionine or AOAA significantly inhibited the JAR cell proliferation induced by 0.25 mmol/L methionine, while in the EBSS and vitamin medium, 2.0 and 2.5 mmol/L ethionine or AOAA significantly inhibited the JAR cell proliferation induced by 2.0 mmol/L methionine (Figure S5F). Consistent results were obtained in Ishikawa and pTr cells treated with methionine, ethionine and AOAA (Figure S5G,H). These results suggested that the methionine transsulphuration pathway plays an important role in promoting cell proliferation.

To further determine the specific effects of methionine on the cell cycle, we performed flow cytometry to quantify the numbers of cells in the G0/G1, S and G2M phases. Compared with 0.25 mmol/L methionine supplementation, the methionine‐free group and the ethionine or AOAA groups treated with methionine exhibited a significant decrease in the number of cells in S phase (Figure 7C). Ishikawa and pTr cells also showed similar results (Figure S6A,B). Collectively, these results suggested that proper methionine levels had a positive influence on cell proliferation and that the methionine transsulphuration pathway plays a critical role during the S phase, which is mainly characterized by DNA synthesis. Furthermore, we used 5‐ethyl‐2'‐deoxyuridine (EDU) to analyse DNA synthesis. As shown in Figure 7D and Figure S6C, methionine supplementation significantly increased DNA synthesis, while no difference was observed between methionine treatment in the presence of ethionine and AOAA and no methionine supplementation.

To verify the effects of CBS on cell proliferation, we determined the impact of methionine on cell proliferation when CBS was silenced by siRNA. The knockdown efficiency was detected by Q‐PCR (Figure S6D). To determine the effects of CBS on the cell cycle, we performed flow cytometry and found that CBS^−/−^ significantly reduced the number of cells in S phase compared with the control (Figure 7E; Figure S6E,F). In addition, DNA synthesis was decreased in CBS^−/−^ cells (Figure 7F; Figure S6G). These results indicated that CBS was critical for DNA synthesis and cell proliferation. Analysis of the relative mRNA expression of methionine metabolism enzymes *MAT2A* showed that the expression of *MAT2A* was significantly decreased in CBS^−/−^ cells (Figure S7A). In summary, using siRNA technology to silence the expression of CBS, we confirmed that the key enzyme in the transsulphuration pathway, CBS, was crucial to promote cell proliferation.

**FIGURE 8:**
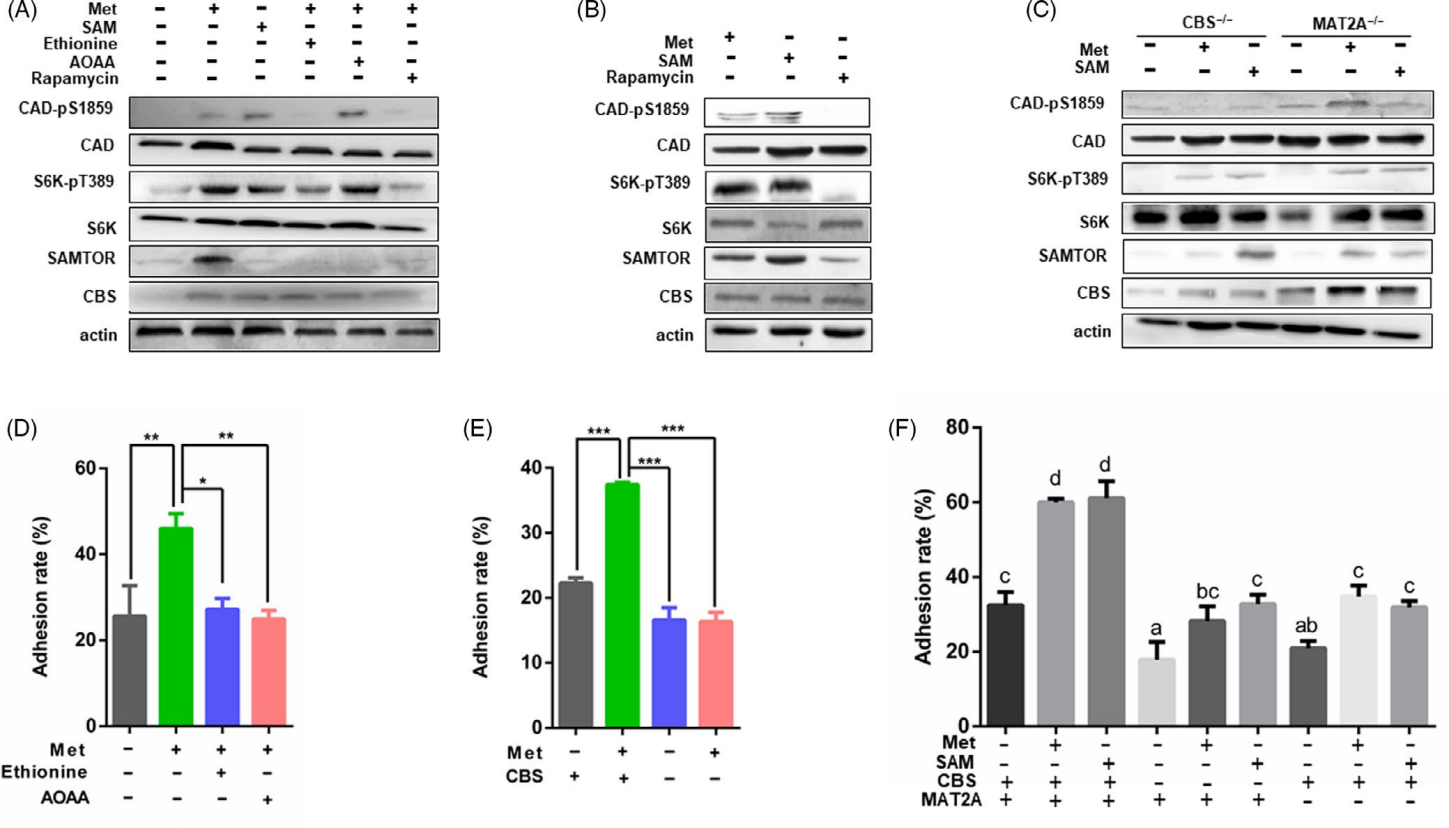
Methionine promoted embryo proliferation and implantation. A, JAR cells were starved overnight in serum and methionine‐free medium. Cells were treated with methionine, SAM, ethionine, AOAA and rapamycin, and then collected for Western blot analysis. B, JAR cells were pre‐treated with rapamycin for 12 h in order to inhibit mTORC1. Afterwards, the culture medium was discarded and cells were rescued with methionine and SAM. Cells were collected for Western blot. C, CBS or MAT2A was silenced in JAR cells and then treated with methionine and SAM. Cells were collected for Western blot analysis. D, JAR and Ishikawa cells were starved overnight in serum and methionine‐free medium and then treated with methionine, SAM, ethionine, AOAA, (E) CBS silence and (F) methionine, SAM, MAT2A/CBS silenced. Then, JAR cells were stained with CFSE and plated on an Ishikawa monolayer cells. Implantation rates were measured using flow cytometry. n = 3. ***P < .001; **P < .01; *P < .05

In addition to CBS, we identified another enzyme, MAT2A, that regulates methionine metabolism and interacts with CBS. To further determine the mechanism of the effect of methionine on embryonic and uterine cell proliferation, our results indicated that methionine and SAM supplementation significantly increased the expression of CBS, SAMTOR, CAD‐pS1859 and S6k‐pT389, while ethionine and AOAA supplementation decreased the expression of CBS, SAMTOR, CAD‐pS1859 and S6k‐pT389 in Ishikawa and pTr cells (Figure 8A). Rapamycin is an inhibitor of mTORC1, and in our experiments, mTORC1 activity was significantly decreased by rapamycin treatment. To confirm the effects of methionine and SAM, we pre‐treated cells with rapamycin to inhibit mTORC1 activity and then supplemented the cells with methionine and SAM. The results showed that methionine and SAM rescued the expression of SAMTOR, CAD‐pS1859 and S6K‐pT389 (Figure 8B). These results indicated that methionine and SAM activate mTORC1 through SAMTOR. Additionally, our results revealed that CBS silencing significantly decreased the expression of SAMTOR, CAD‐pS1859 and S6K‐pT389 (Figure 8C). Compared with the methionine‐free condition, methionine and SAM supplementation increased the expression of these proteins in Ishikawa and pTr cells. These results indicated that CBS plays a critical role in the process by which methionine activates mTORC1. Next, we explored whether the specific active molecule is methionine or its functional metabolite, and our results showed that MAT2A silencing significantly decreased the expression of SAMTOR, CBS, CAD‐pS1859 and S6K‐pT389 (Figure 8C).

### 3.10 Methionine levels affected embryo adhesion in vitro

The Ishikawa cell line was selected to study the effects of methionine on embryo adhesion. The endometrial cell monolayer and JAR cells were pre‐treated with methionine or methionine supplemented with ethionine or AOAA for 12 hours. Then, JAR cells were labelled using CFSE and plated on an Ishikawa monolayer. After 30 minutes, the attached JAR cells were observed under a fluorescence microscope. The results showed that compared with the no treatment, ethionine or AOAA treatment, methionine treatment upregulated the adhesion rate (Figure 8D; Figure S7B). Similarly, the adhesion rate was significantly decreased in the CBS silencing treatment compared with the CBS^+/+^ group (Figure 8E; Figure S7C). Moreover, the adhesion rate was significantly decreased in the MAT2A silencing treatment compared with the control group, and SAM supplementation rescued the embryo adhesion rate (Figure 8F). These results indicated that the methionine metabolism enzymes MAT2A and CBS are crucial for embryo implantation.

## 4 DISCUSSION

Maternal methionine status has many effects during pregnancy. Methionine imbalance harms the short‐term reproductive function of animals and the physiological functions of offspring. Excess methionine results in an increase in serum Hcy levels and leads to a series of pregnancy complications, including pre‐eclampsia, spontaneous abortion, placental abruption and recurrent pregnancy loss.^38^ However, a relatively low maternal methionine intake induces a high rate of foetal neural tube defects in women during pregnancy.^39^, ^40^ Additionally, foetal growth retardation was observed in animal models with restricted methionine intake during pregnancy.^41^ However, to date, the mechanisms of the effects of methionine on early embryo development and embryo implantation have remained largely unknown.

Here, we confirmed that a methionine‐free diet impaired embryo implantation and decreased live litter size, while a proper maternal dietary methionine supply during early pregnancy significantly increased the number of embryo implantation sites and ultimately the live litter size. These data strongly support that maternal methionine supplementation is useful for increasing early embryo survival during early pregnancy, which is consistent with the high concentration of serum progesterone.^42^ Additionally, we found a critical relationship between maternal methionine metabolism and its impact on the development of the embryo and uterus during peri‐implantation. The changes in methionine metabolism during human pregnancy were examined by using stable isotopic tracers of methionine, and the results indicated that the rate of methionine transsulfuration was increased during early pregnancy, while the rate of methionine transmethylation was higher during late pregnancy; however, the physiological significance of these changes is not clear.^43^ Here, we performed a serum metabolomic study and examined the free amino acid in serum concentration by HPLC‐MS/MS. We discovered that the availability of dietary methionine strongly influenced its upstream and downstream metabolites in serum during peri‐implantation. The methionine‐free diet decreased the concentrations of most amino acids, while the methionine supplementation diet increased the concentrations of most free amino acids in serum. The changes in serine, glycine and other metabolite concentrations indicated that maternal methionine levels might influence methionine metabolism during peri‐implantation. Furthermore, we evaluated the key enzymes involved in methionine metabolism and found that the serum CBS concentration was significantly increased in rats fed the 1% methionine diet compared with rats fed the control diet, and the concentrations of transsulfuration pathway end products, including taurine and GSH, were also significantly increased. However, the concentrations of CBS, taurine and GSH were significantly reduced in the methionine‐free diet group compared with the control group. Consistent with this finding, serum metabolomic analysis showed that maternal methionine supplementation increased the levels of metabolites involved in the metabolism of biotin, taurine, hypotaurine, nicotinate, nicotinamide and GSH. Based on these results, we concluded that maternal methionine supplementation enhanced the transsulfuration pathway and improved antioxidant capacity during peri‐implantation, which could be beneficial for embryo implantation.

The expression pattern of amino acid transporters is an important indicator of local amino acid absorption and utilization. Many different types of amino acid transporters are highly expressed during early embryonic development.^31^, ^44^, ^45^, ^46^ Our data demonstrated that the expression of methionine transporters was much higher in implantation sites than in ovarian tissues in rats fed a 1% methionine diet, indicating that methionine might play a much more important role in the implantation sites than in the ovaries. The implantation site includes the embryo and uterine tissue. Thus, we tried to explore the mechanism in these two locations. On the one hand, we found that the expression of key enzymes in methionine metabolism and the concentration of GSH were significantly increased in response to proper methionine supplementation using in vitro pregnant uterus explant cultures separated from rats on day 5 of pregnancy. On the other hand, methionine supplementation significantly impacted the cell cycle, embryonic development and endoplasmic reticulum stress of pTr cells. Notably, we discovered that CBS and MAT2A were of great importance in the regulation of embryonic development.

Endoplasmic reticulum stress can be caused by oxidative stress, resulting in defective embryo development or embryo damage. ROS, which cause oxidative stress, may originate from embryonic metabolism and embryonic surroundings.^32^ The deleterious effects of ROS can alter the development of mammalian embryos in vitro^47^; for instance, the 2‐cell mouse embryo block is associated with an increase in ROS.^47^, ^48^ As the end products of the transsulphuration pathway of methionine, GSH and taurine have strong antioxidant capacities, these substances can reduce the formation of ROS, ultimately protecting embryos against oxidative stress.^49^ Our in vivo data indicated that the GSH, taurine and hypotaurine pathways were markedly strengthened in the 1% methionine group compared with the control group. In addition, the concentration of GSH in the implantation sites of uterine tissues and in pregnant uterus explant cultures was markedly increased in the presence of methionine. Similarly, our in vitro results demonstrated that the ROS levels in JAR, pTr and Ishikawa cells were drastically decreased in the presence of methionine in the culture medium but significantly increased in the presence of a methionine inhibitor, a CBS inhibitor and CBS siRNA. Therefore, our results indicated that methionine exerted its action through the antioxidant capacities of the transsulphuration end products.

The metabolism of early mammalian embryos is very similar to that of rapidly proliferating cells,^50^ which have different metabolic needs than non‐proliferating cells. Methionine is consumed at a higher rate than other essential amino acids in the mammalian cellular transition from quiescence to proliferation^51^; however, the biological role of methionine in embryo development is unclear. mTORC1 is a master regulator of cell growth and anabolic and catabolic processes in response to amino acid signals^52^ and plays a critical role in embryo development. The amino acid sensing pathway upstream of mTORC1 is complicated. Leucine and arginine are well‐established activators of mTORC1 signalling; the transmembrane protein SLC38A9 is a lysosomal arginine sensor, while Sestrin2 and CASTOR1 are cytosolic leucine and arginine sensors, respectively.^53^, ^54^ Our previous studies showed that N‐carbamylglutamate, an analog of endogenous N‐acetyl‐glutamate (an activator of arginine synthesis), enhances pregnancy outcomes in rats by activating the PIK3/PKB/mTOR signalling pathway.^55^ However, whether and how methionine affects mTORC1 signalling is unclear. A recent work identified a new protein, SAMTOR, which binds to SAM, that can inhibit mTORC1 signalling by interacting with GATOR1, the GTPase‐activating protein for Rag A/B.^56^ Here, we revealed that unlike leucine and arginine, methionine sensed mTORC1 indirectly through the functional metabolite SAM under the regulation of MAT2A, which catalyses the production of SAM from methionine and ATP.^57^ Notably, a relationship between MAT2A and CBS was found. The transsulphuration pathway regulated by CBS plays an important role in the catabolic metabolism of Hcy, a potent competitive inhibitor of transmethylation reactions. Both an increase in SAH and a decrease in the SAM:SAH ratio are known to inhibit transmethylation reactions^58^ and the expression of MAT2A. Most recent studies have shown that in addition to protein and lipid/sterol biosynthesis,^59^ mTORC1 signalling promotes DNA synthesis through the post‐translational regulation of S6K1, which directly phosphorylates S1857 on CAD, the enzyme that catalyses the first three steps of de novo pyrimidine synthesis.^60^, ^61^, ^62^ In our study, purine metabolism, DNA replication and cell cycle transition were enhanced in the methionine‐supplemented group. Furthermore, we uncovered that the downstream targets of mTORC1 activated by methionine were S6K1 and CAD, which ultimately improved embryo development and implantation. The mTORC1 pathway has emerged as a critical growth regulatory node that is deregulated in a variety of diseases, including cancer, diabetes and epilepsy. While we found that methionine can be a new activator of mTORC1, many other effects of methionine remain to be explored.

Methionine is an essential amino acid, but the importance of methionine in embryonic development and the underlying molecular mechanisms are widely neglected. Our study uncovered a function and mechanism of methionine in improving embryo development and implantation. Improving our understanding of this field has high clinical and productive significance in natural and assisted reproduction.

## CONFLICT OF INTEREST

The authors declare no competing interests.

## AUTHOR CONTRIBUTIONS

SC and XZ conceived the study and designed experiments; SC, QY, XZ, GY, CY and MC performed the experiments; HY, YW, SH and GW performed transcriptomic data analysis; SC and SQ wrote the paper; XZ and SQ supervised the entire project.

## Supporting information

Fig S1

Fig S2

Fig S3

Fig S4

Fig S5

Fig S6

Fig S7

Table S1‐S2

## Data Availability

The data that support the findings of this study are available from the corresponding author upon reasonable request.
