# Supplementary figures and images for: CBS and MAT2A improve methionine‐mediated DNA synthesis through SAMTOR/mTORC1/S6K1/CAD pathway during embryo implantation
Source: Cell Prolif. 2020 Nov 12;54(1):e12950. doi: 10.1111/cpr.12950 (PMC7791180; doi:10.1111/cpr.12950)

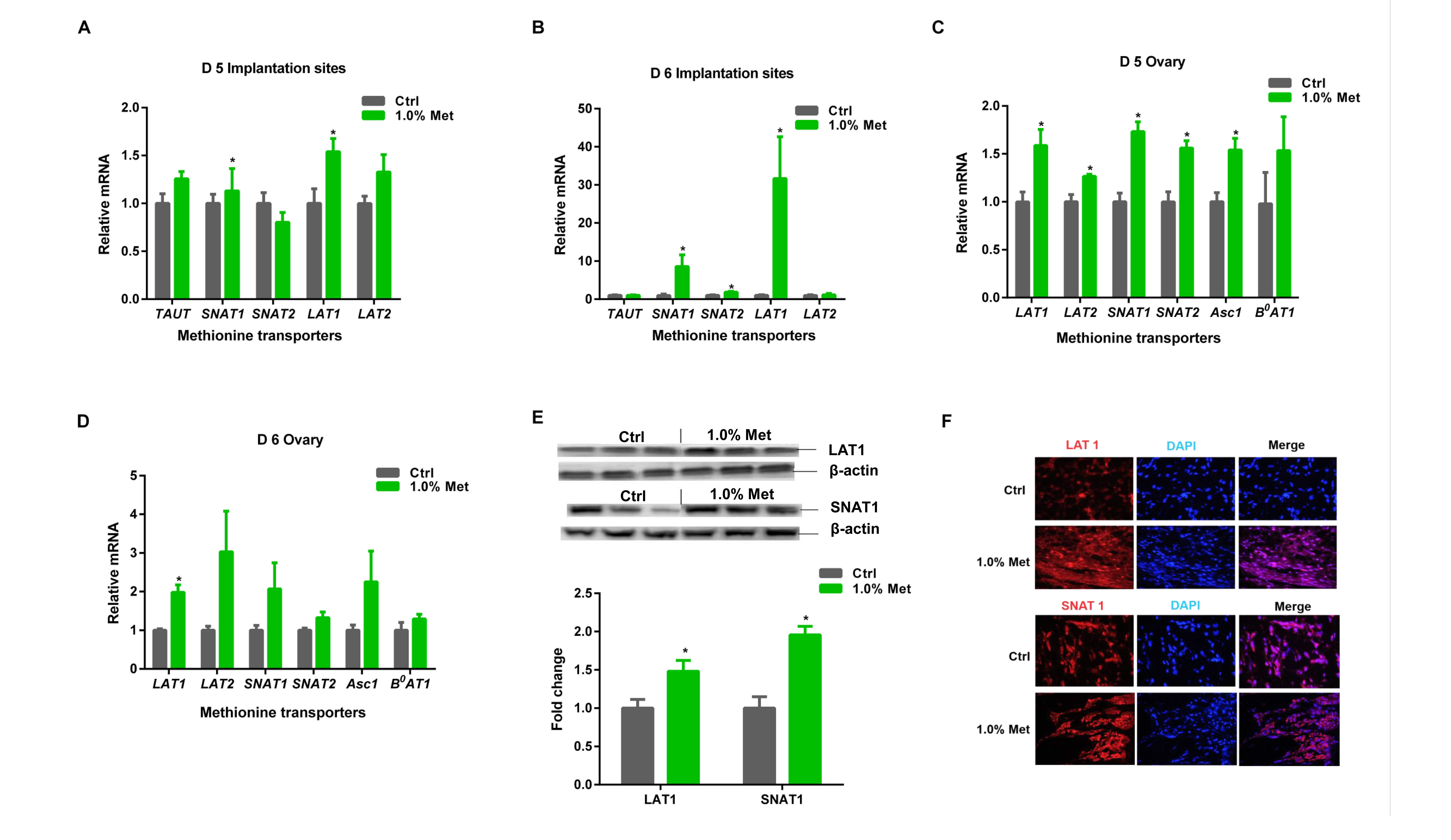

Supplement: Supplementary file 2 — Fig S2 [file CPR-54-e12950-s002.tif]

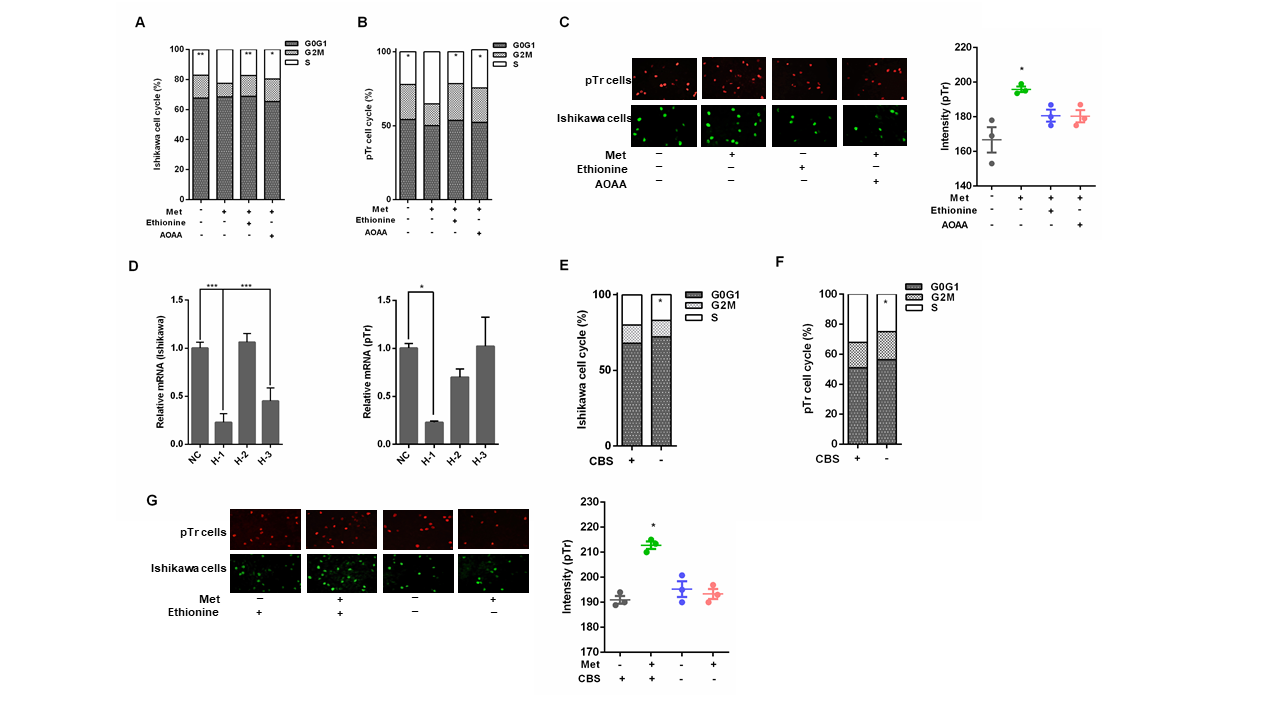

Supplement: Supplementary file 6 — Fig S6 [file CPR-54-e12950-s006.tif]

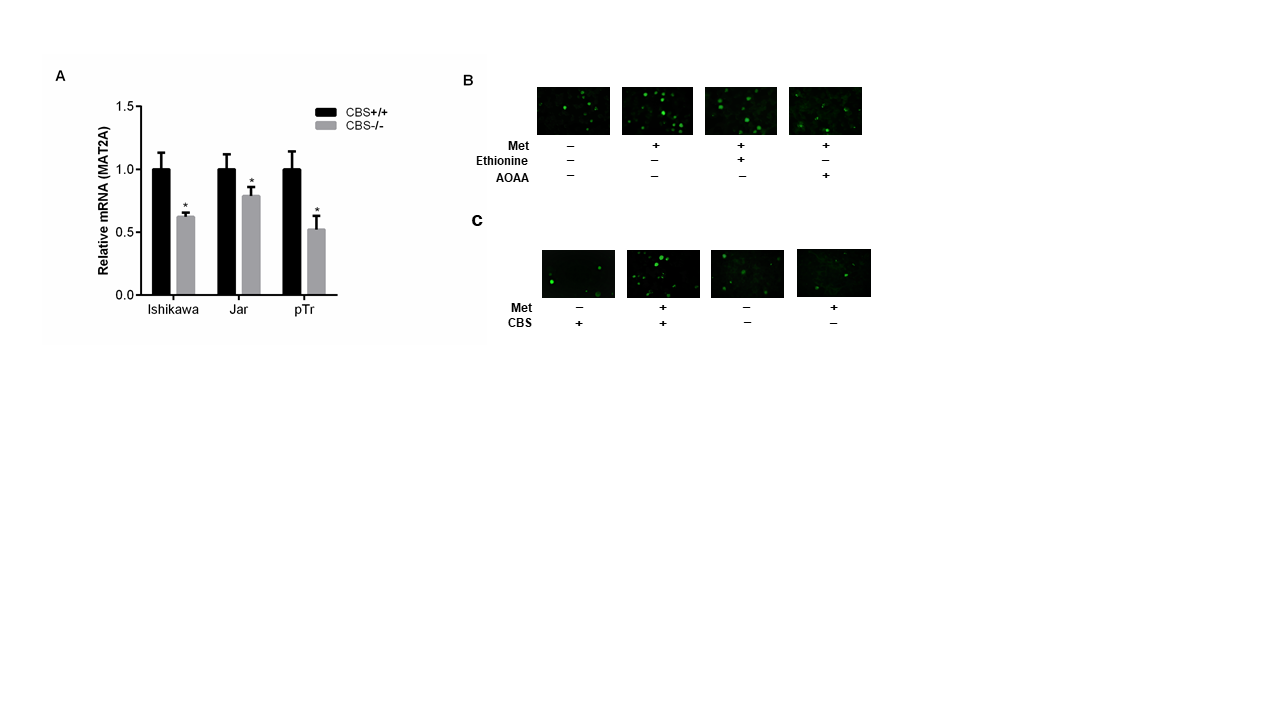

Supplement: Supplementary file 7 — Fig S7 [file CPR-54-e12950-s007.tif]
