# Supplementary material for: CBS and MAT2A improve methionine‐mediated DNA synthesis through SAMTOR/mTORC1/S6K1/CAD pathway during embryo implantation
Source: Cell Prolif. 2020 Nov 12;54(1):e12950. doi: 10.1111/cpr.12950 (PMC7791180; doi:10.1111/cpr.12950)
Supplement: Supplementary file 8 — Table S1‐S2 [file CPR-54-e12950-s008.docx]

**Supplemental Figures/Tables**

**Table S1. PCR primers used for gene expression analysis**

| Gene | Forward primer | Reverse primer |
| --- | --- | --- |
| *LAT1* | GAGACCTCAGAAAAGCCCCT | GGTGAGCTAGGTGGGAAGTT |
| *LAT2* | GAGACCTCAGAAAAGCCCCT | GGTGAGCTAGGTGGGAAGTT |
| *SNAT1* | TTCTTCACAGTCCGGTCCTC | GTCACCCCTACGACTCCAAA |
| *SNAT2* | CTGTCCCCGTCGTCATTTTC | GAACCCGAAGATGTCCCTGA |
| *B0AT1* | GGCTCCATCCCTCTGCTAAT | TCCTCATAGTCAGGGTCCCA |
| *Asc1* | CTTGGTGTTCTGGGCATTCC | AAACCACGAAACACAGCTCC |
| *TAUT* | AACTGCTACATACCTACTCTG | CGGAACCAACTGAACTCTT |
| *CBS* | acacagtgccgacaaaatcc | cagcgtcttcaatcatccgg |
| *Dnmt1* | gtgttttctggctgtggaggc | cacacagcatctccacatcg |
| *Dnmt3* | aggggcatcactggaaatga | agtgaggaagcagtggagac |
| *CSE* | ttcaggaatgggatggcagt | gaagctcagccagactctca |
| *MS* | tggcatgaaagtggttggtg | agggtcctcttcttcaaccg |
| *β-actin* | caaccttcttgcagctcctc | gacccatacccaccatcaca |

**Table S2. SiRNA sequences used for CBS interference.**

| Items |  |  |
| --- | --- | --- |
| Swine |  |  |
| S-1 | GCCAGGAAGUGCCCUGGAUTT | AUCCAGGGCACUUCCUGGCTT |
| S-2 | GCAACGACGAGGAGGCCUUTT | AAGGCCUCCUCGUCGUUGCTT |
| S-3 | GCCCGGGAUGCAAGAUCAUTT | AUGAUCUUGCAUCCCGGGCTT |
| S-NC | UUCUCCGAACGUGUCACGUTT | ACGUGACACGUUCGGAGAATT |
| Human |  |  |
| H-1 | GACUCAGUGCGGAACUACATT | TTCUGAGUCACGCCUUGAUGU |
| H-2 | GUCAGACCAAGUUGGCAAATT | TTCAGUCUGGUUCAACCGUUU |
| H-3 | GGAAGAAGUUCGGCCUGAATT | TTCCUUCUUCAAGCCGGACUU |
